# Impact of health education on internet addiction, internet use time, and social media addiction in adolescents: a systematic review and meta-analysis

**DOI:** 10.1186/s12889-025-25793-4

**Published:** 2025-12-03

**Authors:** Andrés Arana-Rodríguez, Almudena Garrido-Fernández, Miriam Sánchez-Alcón, Julia Sánchez-Galloso, Álvaro-José Rodríguez-Domínguez, Francisca María García-Padilla

**Affiliations:** 1 Department of Nursing, University of Huelva, Huelva, 21071 Spain; 2 Department of Health and Sports, Pablo de Olavide University, Seville, 41013 Spain; 3 Nursing Faculty, Nursing Department, University of Huelva, Avenida Tres de Marzo, s/n, Huelva, 21071 Spain

**Keywords:** Adolescent, Health education, Health promotion, Internet addiction disorder, Behavior, Addictive

## Abstract

**Background:**

Internet and social media addiction have become a health problem due to their negative impact on health and well-being. Internet use time is also frequently examined as an outcome, offering complementary insight into usage patterns. Health education is a key tool during adolescence to promote healthy behaviours, supporting self-regulation and fostering healthier patterns of Internet and social media use. The aim of this study is to evaluate the effectiveness of health education interventions for reducing Internet addiction, Internet use time, and social media addiction among adolescents.

**Methods:**

We conducted a systematic review with meta-analysis following PRISMA guidelines. Eligible studies were experimental or quasi-experimental designs involving adolescents and evaluating health education interventions, with outcomes on Internet addiction, Internet use time, or social media addiction. A search was conducted in Medline, WoS, Scopus, CINAHL, PsycINFO and PubMed Central. Study selection, data extraction, and synthesis were performed independently by pairs of researchers. To evaluate risk of bias, the Cochrane Collaboration’s tool was used for randomised studies, and ROBINS-I for non-randomised studies. Certainty of evidence was assessed with GRADE.

**Results:**

Sixteen studies with 13,562 participants were included in the systematic review. Six were included in the meta-analysis for Internet addiction. Most interventions were conducted in educational settings, with participants showing variability in their Internet use patterns across studies. Across studies, observed characteristics included group delivery and participatory approaches, parental involvement, promotion of healthier Internet use, and inclusion of broader educational content addressing health-promotion topics. The review found generally favourable effects on Internet addiction and Internet use time. Evidence on social media addiction was limited. The meta-analysis for Internet addiction showed a statistically significant difference in favour of the intervention, with a large effect size (SMD = − 1.88; 95% CI [− 2.64, − 1.11], *p* < 0.00001). Subgroup analysis showed a large and significant effect in adolescent only interventions (SMD = − 1.93; 95% CI [− 2.84, − 1.02], *p* < 0.0001) and in parent involved interventions (SMD = − 1.81; 95% CI [− 2.95, − 0.68], *p* = 0.002).

**Conclusions:**

Health education shows promise for reducing Internet addiction and Internet use time among adolescents. Evidence on social media addiction remains limited.

**Trial registration:**

The study protocol was registered in PROSPERO (CRD42024568029).

## Background

Internet addiction (IA) is defined as the uncontrolled use of the Internet that negatively impacts quality of life in the physical, psychological, and social spheres. It is also characterised by the compulsive use of various digital online media with social functions, online shopping, or online video games, among others, and includes the appearance of withdrawal symptoms, a negative impact on families, and significant financial expenditure derived from online activities [1–3].

Social media can be defined as “Internet-based channels that allow users to opportunistically interact and selectively self-present, either in real-time or asynchronously, with both broad and narrow audiences who derive value from user-generated content and the perception of interaction with others” [4].

Social media addiction (SMA), meanwhile, is characterised by a loss of control over social media use, with significant negative consequences for both psychological and social health [5, 6]. SMA has been recently identified as a potential public health concern, with a meta-analysis finding a prevalence of 24% across 32 nations [7].

According to recent research, adolescents with higher levels of addictive behaviours related to social media use are more likely to experience psychosocial health problems than those with lower levels. Moreover, findings showed that greater addictive behaviours are associated with greater psychosocial difficulties in this population [8].

As for IA, its prevalence rate reaches 7.02%, standing at 8.9% in Eastern countries and 4.6% in Western countries [9]. There is no clear trend in terms of gender with regard to IA. However, when focusing on social media, women appear to have higher levels of SMA [10].

In adolescents, Internet addiction has been associated with a variety of negative psychosocial outcomes. A recent meta-analysis found significant associations between Internet addiction and depression, anxiety, aggressiveness, lower psychological well-being, and reduced self-esteem, underlining the impact of IA on adolescent mental health [11].

IA has been described using a variety of terms in different contexts over the last decades [12]. Even so, it was first described as a health issue by Dr. Young [1]. This author also designed what remains the most widely used tool for assessing this phenomenon, the Internet Addiction Test (IAT) [13, 14], despite the multiplicity of scales developed in recent times [15].

Social media are online platforms that encourage reward-seeking behaviour, comparable to a Skinner box, with teenagers tending to be more vulnerable to these stimuli [16, 17]. Reward sensitivity in adolescents has been associated with the physiological changes occurring in their neurodevelopment, which is also related to their tendency towards social interactions with their peers [18, 19].

On the other hand, in adolescents who develop a pattern of Internet addiction, functional changes in the brain have been observed, resembling those derived from substance abuse and gambling addiction, thereby influencing the reward pathway [20]. According to the proposals for defining a diagnosis, the affected person can experience loss of control and tolerance, needing to increase the hours of use and upgrade their technological equipment. They may also suffer social isolation and withdrawal symptoms such as depression or anger, and neglect other responsibilities, such as academic ones [21, 22]. Hence, the time adolescents spend online can be seen as related to tolerance and loss of control, features frequently highlighted in descriptions of addictive behaviour.

The existing interventions to address this issue include cognitive behavioural therapy, pharmacology, family therapy, and physical exercise, among other strategies [23]. Including families and teachers in the interventions is also important for the prevention of IA [24]. This is consistent with the evaluation conducted by Blum and colleagues of effective youth development programmes, which emphasises the importance of parents, schools, and the community in fostering adolescents’ health and well-being [25].

Previous reviews show that interventions to promote digital well-being and reduce problematic technology use for children, adolescents, and young people, were most often delivered in educational settings. They generally included education on digital well-being and media literacy, as well as the fostering of self-regulation and coping skills [26].

Adolescence is also a crucial period for adopting behaviours that can generate long-term health benefits or risks throughout life. During this stage, the developmental changes leave adolescents particularly vulnerable to risk-taking, but also open a window of opportunity for prevention and health promotion. Habits established at this time often persist into adulthood and may even influence the health of future generations [27]. Therefore, health education interventions in adolescence can have a meaningful impact not only in the short term but also in shaping healthier trajectories across the life course.

In this study, the focus will be on health education, which is defined as the building of learning opportunities designed to improve health literacy, knowledge, and skills that increase individual and community health levels [28]. In adolescence, health education is considered a priority, as it provides the necessary tools for making informed decisions about well-being and health [29].

Previous works have examined interventions to prevent or reduce problematic Internet and social media use, but with different scopes. Among them, a school-based prevention review on Internet addiction in adolescents reported that some interventions also addressed related health problems. The review highlighted that risk behaviours appear to be interrelated, suggesting that prevention programmes should address related health issues and incorporate goals such as refusal skills and awareness [30]. A narrative review on problematic social media use and the impact of related interventions on adolescent mental health highlighted the potential of school strategies [31]. In addition, a systematic review with meta-analysis of non-pharmacological interventions in young people with Internet addiction identified educational interventions among the strategies assessed, although only a small number of studies fell into this category [32]. To our knowledge, the specific role of health education interventions in adolescents, covering Internet addiction, social media addiction and Internet use time, has not yet been synthesised through a systematic review with meta-analysis, which is the aim of the present study.

## Methods

### Search strategy

A search was conducted following the PRISMA guidelines [33]. The protocol was registered in PROSPERO. The search process was carried out from the first publication found on the topic to 1 August 2024. Sources consulted included the bibliographic databases Medline (via PubMed), Web of Science (WoS), Scopus, Cumulative Index to Nursing and Allied Health Literature (CINAHL), and PsycINFO, as well as PubMed Central (PMC), an open-access repository included as part of the ‘other sources’ in the protocol, which contributed substantially to the records initially identified. The search strategy was as follows: (‘Teenagers’ OR ‘Youths’ OR ‘Adolescents’ OR ‘Students’) AND (‘Health promotion’ OR ‘School Health Services’ OR ‘School-based intervention’ OR ‘Educational intervention’ OR ‘Health education’ OR ‘Prevention education’) AND (‘Internet addiction disorder’ OR ‘Problematic use of Internet’ OR ‘Internet addiction’ OR ‘Social media addiction’ OR ‘excessive use of Internet’ OR ‘overuse of Internet’ OR ‘ICT addiction’). This search strategy was adapted using controlled and natural language in the respective thesauri of each search engine.

Additionally, a manual search of the reference lists included in the identified studies was conducted to identify possible additional records.

### Inclusion and exclusion criteria

Articles that met the following inclusion criteria were included: (1) Intervention aimed at the adolescent population, defined as individuals aged 10–19 years according to the World Health Organization [34], with or without the inclusion of their parents; (2) Studies including a health education intervention in any of its modalities applied to the experimental group; (3) Studies stating a comparison with a group that received any intervention, no intervention, or no comparison to another group; (4) Studies whose outcomes regarding Internet addiction, Internet use time, or social media addiction were measured; (5) Experimental or quasi-experimental designs; (6) Studies in Spanish, English, or French.

The exclusion criterion was as follows: Studies not published in scientific journals (e.g. theses, technical reports, conference abstracts without publication in full).

### Screening

Two researchers (A.A-R. and A.G-F.) independently and manually screened the articles based on their titles and abstracts, using the research question and applying the inclusion and exclusion criteria. Next, the pre-selected articles were read in full to assess whether they met the eligibility criteria. In case of disagreement, a third researcher was consulted to resolve the issue (M.S-A.). Mendeley software was used to organize and manage the references.

### Procedure and data extraction

A data extraction table was designed based on authors, study design, population intervention and control group, evaluation period, measurement instruments, and main results. Participant characteristics, such as population and sample size, were also included in the table, while the eligibility criteria used in each study are described in the Results section. We extracted information on all evaluation periods and on the measurement instruments used to assess our target variables (Internet addiction, Internet use time, and social media addiction). The main results were summarized in the table to give a clear overview, and further details are provided in the Results section. For the meta-analysis, we selected the first post-intervention assessment, since this was the time point most consistently reported by the studies. Two researchers carried out this task independently (A.A-R. and F.M.G-P.). Any potential disagreements were resolved by consensus through the mediation of a third researcher (A.G-F.).

### Risk of bias assessment

To assess the risk of bias, the Cochrane Collaboration’s tool was used for studies with randomisation [35] and the ROBINS-I tool for those that did not include a randomisation process to determine the experimental and control groups [36]. The *robvis* tool was used for visualisation of ROBINS-I [37] and Review Manager version 5.4.1 for the visualization of The Cochrane Collaboration’s tool. This task was carried out independently by two researchers (A.A-R. and M.S-A.). Any discrepancies were resolved by consensus with the mediation of a third researcher (J.S-G.).

### Data synthesis and analysis

For data analysis, a systematic review was conducted. The qualitative synthesis was performed by two authors (A.A-R. and J.S-G.), and, whenever possible, a meta-analysis was conducted by another author (A.J.R-D.) and verified by a second author (A.A-R.). To this end, the measurements results reported in the included studies were collected, i.e. the means and standard deviations. When studies provided other types of measurements (such as medians or quartiles Q1 and Q3), we calculated estimates to approximate the mean and standard deviation [38]. Because the studies used different tools to assess the ‘Internet addiction’ variable, the inverse variance method was used to calculate the estimated effect and its standard error [39]. A random-effects model was employed for the meta-analysis in cases of heterogeneity. In all other cases, a fixed-effect model was used. I^2^ < 50% and Chi^2^ test values at *p* >0.05 were taken as indicators of homogeneity [40]. To estimate the effect size, the reference values indicated by Cohen in 1988 were used [41], where standardised mean difference (SMD) values between 0.2 and 0.49 are interpreted as a ‘small effect’, between 0.5 and 0.79 as a ‘moderate effect’, and values above 0.79 are interpreted as a ‘large effect’. Review Manager software version 5.4.1 was used for the statistical analysis.

Where possible, publication bias was assessed through Egger’s test and funnel plots [42] using the Epidat 3.1 software. In addition, a sensitivity analysis was performed to assess the degree of influence of each study included in the meta-analysis results. Review Manager version 5.4.1 software was used for this purpose.

### Certainty of the evidence

The Grading of Recommendations Assessment, Development and Evaluation (GRADE) tool was used to assess the certainty of the evidence, specifically the professional GRADE Pro/Guideline Development Tool. This tool allows for the assessment of risk of bias, inconsistency, imprecision, indirect evidence, and publication bias, and classifies evidence according to different levels of confidence [43].

## Results

### Selection process

**Fig. 1:**
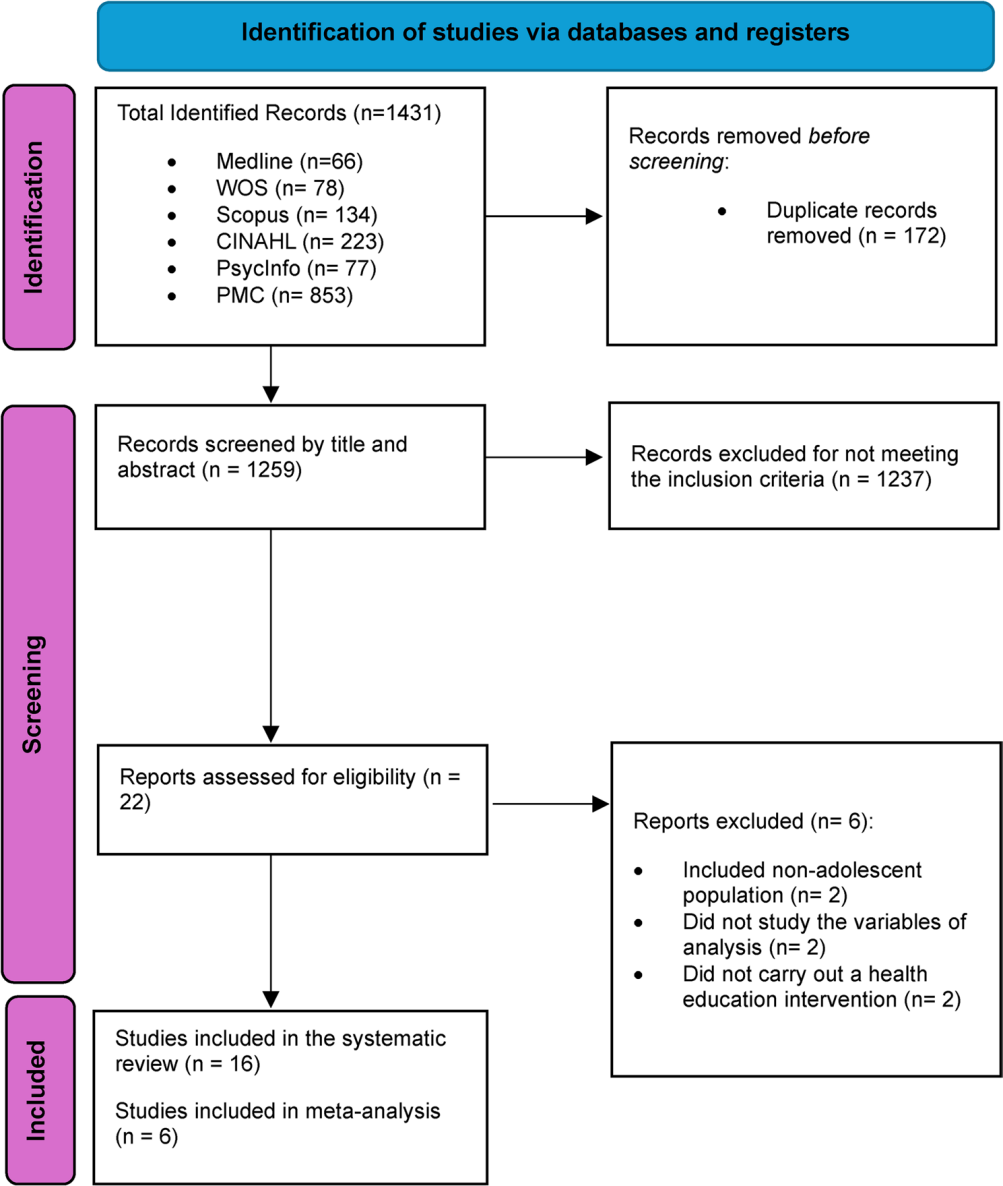
PRISMA flow chart

Figure 1 shows the selection process of studies. A total of 1,431 studies were identified, and after removing duplicates and assessing the inclusion criteria, 16 were finally selected for systematic review and 6 for meta-analysis, as the necessary data for inclusion were provided.

### Characteristics of the included studies

A summary of the studies included is provided in Table 1 was produced. In the systematic review, heterogeneity was found in the types of study designs, which were therefore classified into two main groups based on whether they were experimental or quasi-experimental designs. Seven of the studies used an experimental design [44–50] and nine other studies applied a quasi-experimental design [51–59].

**Table 1:** Characteristics of the included studies

Authors	Design	Population	EG	CG	Evaluation period	Measurement instruments	Main results
Busch et al., 2013 [51]	Non-randomised trial Quasi-experimental	*N* = 356 students Experimental Group (EG): *n* = 136 Age range = 15–16 years Control Group (CG): *n* = 220 Age range = 15–16 years	Cross-sectional implementation during the health education and promotion training course, covering topics such as nutrition, physical exercise, sexual health, and reduction in drug use, sedentary behaviour, bullying, and compulsive behaviour related to the Internet and video games. Its principles include the active involvement of parents in the activities.	Group from the same educational centre before the intervention was implemented	Pre-intervention Post-intervention	Questionnaire including time spent playing online video games CIUS	The experimental group showed a statistically significant decrease in the CIUS scale in adolescent females.
Shek and Ma, 2014 [52]	Randomised trial Quasi-experimental	*N* = 3,328 students (from 28 educational centres) Mean age = 12.59 ± 0.74 years EG: *n* = 16 educational centres CG: *n* = 12 educational centres	Tier 1 intervention: 10–20 h in sessions focused on 15 constructs of positive youth development: bonding, resilience, social competence, emotional competence, cognitive competence, behavioural competence, moral competence, cultivation of self-determination, spirituality, development of self-efficacy, development of a clear and positive identity, promotion of beliefs in the future, provision of recognition for positive behaviour, provision of opportunities for prosocial involvement, and fostering prosocial norms. Tier 2 intervention: The school social work agency collaborated with the school to develop the tier 2 intervention using positive youth development constructs.	Did not participate in the project.	4 months after the school term commenced	IAT	The experimental group did not obtain statistically significant differences for Internet addiction.
Walther et al., 2014 [44]	Randomised trial Experimental	*N* = 1843 students EG: *n* = 804 Mean age = 11.8 ± 0.8 years CG: *n* = 1039 Mean age = 12.1 ± 0.83 years	Four 90-minute sessions, implemented by teachers, on the use of the Internet, online communication, gaming, and gambling. The intervention also contained a booklet for parents that provided recommendations.	Attended regular classes.	Pre-intervention Post-intervention Follow- up: 12 months	ISS Questionnaire that includes frequency of Internet use in days per month, percentage of daily users, Internet use in hours per day, and percentage of excessive Internet use per day.	A significant group–time interaction for the German Internet Addiction Scale (ISS), showing a marked increase in the scale for the control group.
Çelik, 2016 [53]	Randomised trial Quasi-experimental	N= 30 students (adolescents in secondary education) EG: n=15 CG: n=15	Five weekly session of 90–120 minutes at the educational centre, focused on reducing Internet usage time and increasing conscious Internet use, academic motivation, and efficient use of time, and on reducing Internet addiction.	Did not receive the intervention.	Pre-interventionPost-interventionFollow-up: 6 months after the intervention.	PIUS	The experimental group achieved statistically significant improvements compared to the control group in the PIUS.
Ruggieri et al., 2016 [54]	Non-randomised trial Quasi-experimental	N=90 students EG “A”: n=45 male students Age = 13 years EG “B”: n=45 female students Age = 13 years	EG ‘A’ Weekly 3-hour sessions for one year. The aim was to promote attitudes, habits, and healthy living behaviours in relation to the use of the Internet. The intervention involved the use of active methods such as brainstorming, circle time, role playing, tutoring, or peer action. EG ‘B’ Received the same intervention.	No control group.	Pre-intervention Post-intervention	IAT	A significant positive difference was found in the post-treatment IAT values for both males and females. No statistically significant differences were found when comparing the group of male students with the group of female students.
Manwong et al., 2018 [45]	Randomised trial Experimental	N=244 students EG: n=124 Age range = 12–14 years CG: n=120 Age range = 12–15 years	One 45–50-minute group session per week for 8 weeks. It was divided into three phases: Phase I (health education and feedback), Phase II (commitment and strengthening), and Phase III (follow-through).	Attended regular classes.	Pre-intervention Post-intervention Follow-up: 1 month after the intervention.	S-MAT Questionnaire on average time spent on social media during the week and at weekends.	The experimental group showed a statistically significant decrease in social media usage time in the post-intervention and one month later.
Yang and Kim, 2018 [55]	Non-randomised trial Quasi-experimental	N= 79 EG: n=38 Age range = 13–15 years CG: n=41 Age range = 13–15 years	Ten 45-minute sessions per week in small groups. Methodologies such as empathic communication, peer support, health education, role modelling, case observation, video viewing, praise, feedback, counselling, and physical activity were used.	Did not receive the intervention.	Pre-intervention Post-intervention	K-scale Survey on Internet usage time in minutes per day during the week and at weekends.	Internet addiction and Internet usage time significantly decreased in the intervention group compared to the control group.
Uysal and Balci, 2018 [46]	Randomised trial Experimental	N= 84 EG: n=41 Age range = 11–16 years CG: n=43 Age range = 11–16 years	Eight 40–80-minute training sessions over a 3-month period. Topics such as healthy Internet use, the effects of Internet addiction on social life, sedentary lifestyles, and Internet use were addressed, and awareness was raised about setting goals and achieving change. Activities included watching films, playing games, drawing, and designing posters, among others. Parents were also involved in the activities.	Did not receive the intervention.	Pre-intervention Post-intervention Follow- up: 6 months after the intervention.	IAS	The experimental group obtained statistically significant improvements in the post-intervention and follow-up.
Bonnaire et al., 2019 [47]	Randomised trial Experimental	N= 384 Mean age: 13.2±0.5 years EG: n=190 CG: n=194	A 90-minute training session to raise awareness about screen use, reflect on life priorities, raise awareness of the consequences of excessive use of video games, and reinforce protective factors.	Did not receive the intervention.	Pre-intervention Post-intervention Follow- up: 4 months after the intervention.	Questionnaire including the number of minutes spent online per day during the week and at weekends.	The experimental group reported statistically significantly less Internet usage time at 4 months than the control group.
Gholamian et al., 2019 [56]	Randomised trial Quasi-experimental	N=120 EG: n=60 Age range = 16–17 years CG: n=60 Age range = 16–17 years	A preliminary session for students and parents on Internet addiction and its consequences. This was followed by two sessions for students and one session for mothers, with activities such as discussion groups, encouraging sports, brainstorming, videos, and role-play.	Did not receive the intervention.	Pre-intervention Post-intervention	Questionnaire including Internet usage time in hours per day.	Internet usage time in the experimental group decreased significantly compared to the control group.
Tang et al., 2021 [48]	Randomised trial Experimental	N=775 EG: n=327 Mean age = 15.37±1.31 years CG: n=448 Mean age = 15.32±1.24 years	4 interventions over 2 years with the aim of raising awareness about Internet use, promoting the formation of intention of Internet use behaviour, helping to formulate plans, and strengthening the maintenance of self-efficacy. Health education methodologies, personalised manuals, and guidance on plan development were used, and physical exercise was encouraged.	Did not receive the intervention.	Pre-intervention 6 months 12 months 18 months Post-intervention	Survey including average daily Internet use during the week, at weekends, and at night.	There was a statistically significant decrease in the number of participants who reported using the Internet for more than 4 hours per day at weekends in the experimental group.
Ariyadasa et al., 2022 [57]	Non-randomised trial Quasi-experimental	N= 570 EG: n=280 Age range = 15–19 years CG: n=290 Age range = 15–19 years	3 two-hour training sessions, one week apart. The first module included a lecture and video presentation with questions and answers. The second module addressed types of Internet addiction using PowerPoint and brainstorming. The third module dealt with preventive strategies and responsible use of the Internet. PowerPoint and interactive discussion groups were used regularly.	Did not receive the intervention.	Pre-intervention Post-intervention (12 weeks after the pre-intervention analysis)	IAT Proportion of participants who used the Internet more than 3 hours per day for non-academic use. Proportion of participants with self-perceived excessive use of social media.	The experimental group showed statistically significant improvements compared to the control group in the proportion of adolescents who self-reported excessive social media use and in the proportion of adolescents with Internet addiction. The intervention group significantly improved their mean IAT score compared to their baseline.
Bağatarhan and Siyez, 2022 [58]	Non-randomised trial Quasi-experimental	N=52 10th-grade students EG 1: n=13 EG 2: n=13 CG 1: n=13 CG 2: n=13	EG 2 Eight 90-minute sessions to reduce Internet addiction and change teenagers' perceptions. Presentations, icebreakers, questions and answers, brainstorming, working groups, case studies, assignments, games, and role-play were used, among other methods. EG 1 Four 45-minute sessions for parents were also added to the EG2 education plan, focusing on preventing Internet addiction, improving the relationship with their children, and problem-solving skills. Icebreakers, presentations, questions and answers, group work, worksheets, assignments, and role-play were used.	CG 1 Did not receive the intervention. CG 2 Did not receive the intervention.	Pre-intervention Post-intervention Follow- up: 5 weeks after the intervention.	IAT OCS	Both experimental groups obtained statistically significant improvements in the post-intervention and at 5 weeks compared to the control groups on both the IAT and OCS scales.
Şermet Kaya et al., 2023 [49]	Randomised trial Experimental	N=44 EG: n=22 Mean age = 15.63 ± 0.49 years CG: n=22 Mean age = 15.59 ± 0.50 years	45–60-minute sessions conducted by a nurse specialising in public health. In the first 4 months, 7 weekly face-to-face sessions were held with adolescents and 3 home visits were made to parents. In the following 6 months, there were two face-to-face meetings and one phone call. Topics such as Internet addiction, academic achievement, health, Internet use planning, sleep hours, and academic tasks were addressed.	Did not receive the intervention.	Pre-intervention Post-intervention (first phase) Follow- up: 6 months after the intervention (second phase)	IAT PCIAS	The experimental group obtained statistically significant improvements in the IAT and PCIAS in the post-intervention and follow-up periods.
Favini et al., 2023 [59]	Non-randomised trial Quasi-experimental	N=462 EG: n=248 Age range = 14–17 ± 0.55 years CG: n=214 Age range = 14–17 ± 0.45 years	Four one-hour meetings. The first meeting focused on the promotion of youths’ awareness of smartphone and SNS problematic use. The second addressed behavioural addictions. During the third meeting, a positive and beneficial use of media and ICTs was promoted. The fourth included self-assessment of behaviours and promoted self-efficacy. Discussion methods, games, practical experiences, and self-monitoring were used.	Did not receive the intervention.	Pre-intervention Post-intervention	BSMAS	A significant improvement was found in favour of the intervention in terms of social media addiction.
Otsuka et al., 2023 [50]	Randomised trial Experimental	N = 5,101 (students from 10th to 12th grades) EG: n=2597 CG: n=2504	10 weekly sessions of 10 minutes. The first session was a self-reflection on students’ usage patterns using the K-scale. The next two were lectures on Internet addiction and related health problems. The next two discussed the advantages and disadvantages of Internet and smartphone use. In the next two, the students proposed problem-solving strategies. In the eighth session, the students recorded their Internet or smartphone use in diaries. In the ninth session, the students evaluated their usage patterns through these records. In the last session, the students revised their previous work and planned for future prevention. All this was supported with workbooks.	Did not receive the intervention.	Pre-intervention 2 months post-intervention	K-scale Internet usage time.	There were no statistically significant differences across the groups.

*EG* Experimental Group, *CG* Control Group, *BSMAS* Bergen Social Media Addiction Scale, *CIUS* Compulsive Internet Use Scale, *HAPA* Health Action Process Approach Model, *IAT* Internet Addiction Test, *IAS* Internet Addiction Scale, *ISS* German Internet Addiction Scale, *K-scale* Internet Addiction Proneness Scale, *PIUS* Problematic Internet Use Scale, *OCS* Online Cognition Scale, *PCIAS* Parent–Child Internet Addiction Scale, *P.A.T.H.S.* Positive Adolescent Training through Holistic Social Programs, *S-MAT* Social Media Addiction Test

A total of 13,562 participants had been evaluated in the studies included in the review, with the smallest sample size being 30 [53] and the largest being 5,101 [50]. The adolescent population included in some articles was subject to additional eligibility criteria depending on the study, such as: adolescent population with an IAT score of 50–79 and poor sleep quality [49]; at risk of Internet addiction [58]; considered to be excessive Internet users [48]; score greater than or equal to 90 on the Internet Addiction Scale (IAS) [46]; with Internet use exceeding 8 h per day [56]; population using social media [45]; with the exclusion criterion being adolescents who did not use the Internet [50] or if they scored higher than 108 on the Internet Addiction Proneness Scale [55], among others.

Different educational interventions were applied, with varying durations and frequencies, ranging from a single session [47] up to 10 sessions [50, 55]. The length of sessions ranged from 10 min [50], to 120 min [53, 57], and up to 180 min [54]. In addition, some educational centres included health education in a cross-curricular manner in their daily activities, without a defined duration [51]. The most common range was between 40 and 90 min per session [44–47, 49, 55, 58].

Most interventions were conducted with secondary or high-school students in educational settings, delivered in person and in group formats. Across the included studies, facilitators were generally described as educational staff, healthcare and prevention professionals. One intervention was implemented from a public-health perspective by a nurse, combining school meetings with home visits [49].

On the whole, the sessions covered topics related to reducing excessive Internet use and promoting healthier Internet habits, and were supported by participatory, skills-building approaches, including role-play, brainstorming, group discussions, and worksheets. Also, some studies complemented these educational sessions with additional health promotion content [45–49, 51–56, 58] and others also involved parents in the intervention [44, 46, 49, 51, 56, 58]. The interventions commonly aimed to increase adolescents’ awareness of Internet risks, strengthen their self-regulation skills, support problem-solving, and encourage healthier Internet-use habits.

A total of 11 studies measured Internet addiction, nine measured Internet usage time, and two measured social media addiction [45, 59]. The most common assessment times were pre-intervention and post-intervention, with follow-ups ranging from one month [45] up to 12 months [44]. The IAT, IAS, Compulsive Internet Use Scale (CIUS), German Internet Addiction Scale (ISS), Problematic Internet Use Scale (PIUS), Internet Addiction Proneness Scale (K-scale), Online Cognition Scale (OCS), Parent–Child Internet Addiction Scale (PCIAS), were used to measure Internet addiction, with the IAT being the most frequently used tool [49, 52, 54, 57, 58]. A variety of questionnaires were also used to measure Internet usage time, generally referring to overall Internet use, although in some cases focusing on specific activities such as social media or online gaming. The Bergen Social Media Addiction Scale (BSMAS) and Social Media Addiction Test (S-MAT) were used to measure social media addiction [45, 59].

Statistically significant differences were found in nine studies that measured the ‘Internet addiction’ variable in at least one of the assessment processes [44, 46, 49, 51, 53–55, 57, 58]. In the studies that measured the ‘Internet use time’ variable, six found significant differences in some of their measurement outcomes [45, 47, 48, 55–57]. Finally, one study that assessed the ‘Social media addiction’ variable found significant differences [59]. In addition, the six studies that also included parents in their interventions achieved statistically significant improvements in at least one of the variables analysed [44, 46, 49, 51, 56, 58]. Despite the heterogeneity of health education interventions, the effective interventions tended to encourage active participation, foster practical skills, promote healthier patterns of Internet use, include broader health-promotion topics, and be delivered mainly in educational settings, with some also involving parents to varying degrees.

### Risk of bias and quality assessment

**Fig. 2:**
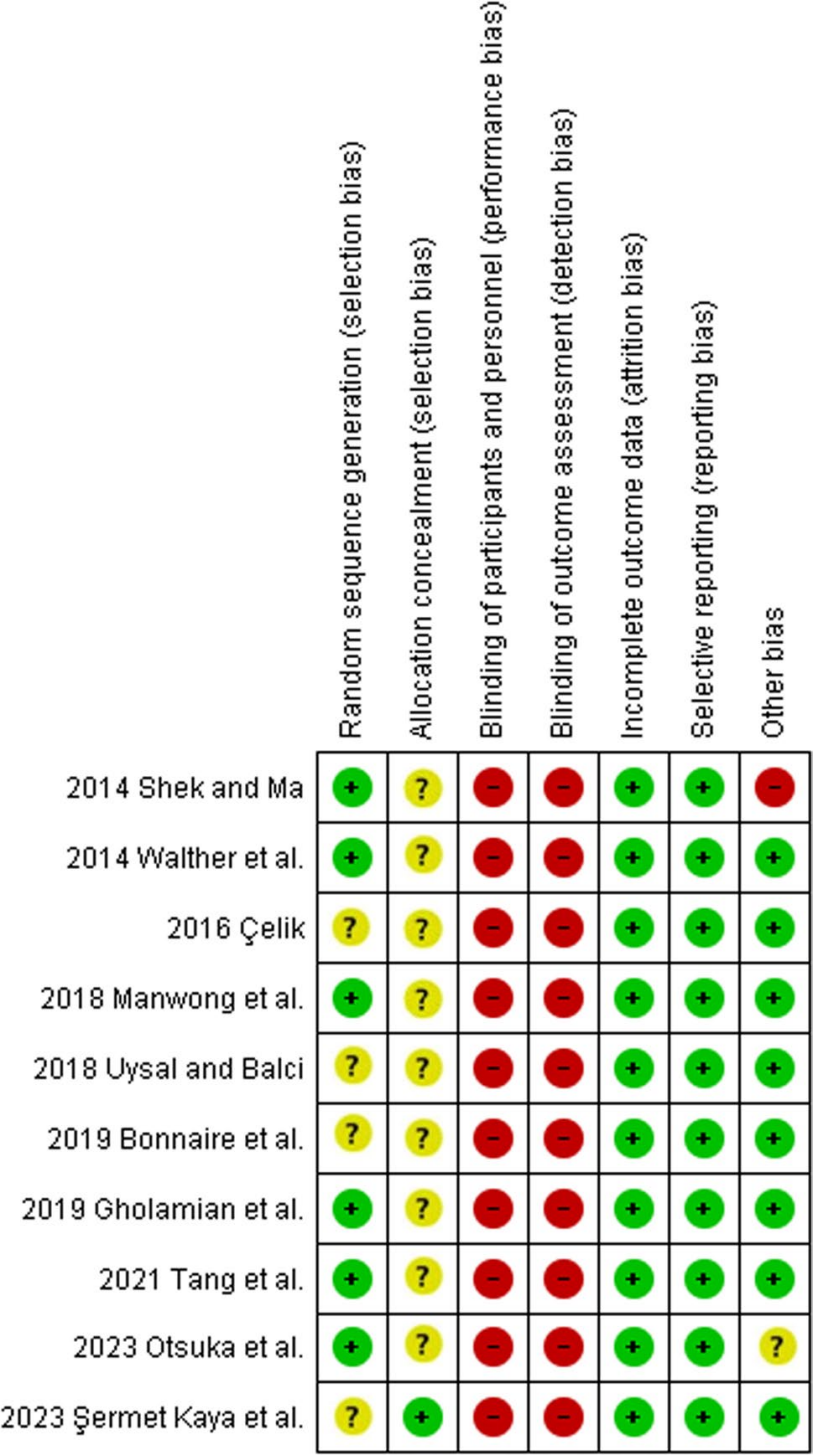
Risk of bias assessment of the randomised controlled trials using the Cochrane Collaboration’s tool. Judgements are shown for each domain. Green = low risk; yellow = unclear risk; red = high risk

**Fig. 3:**
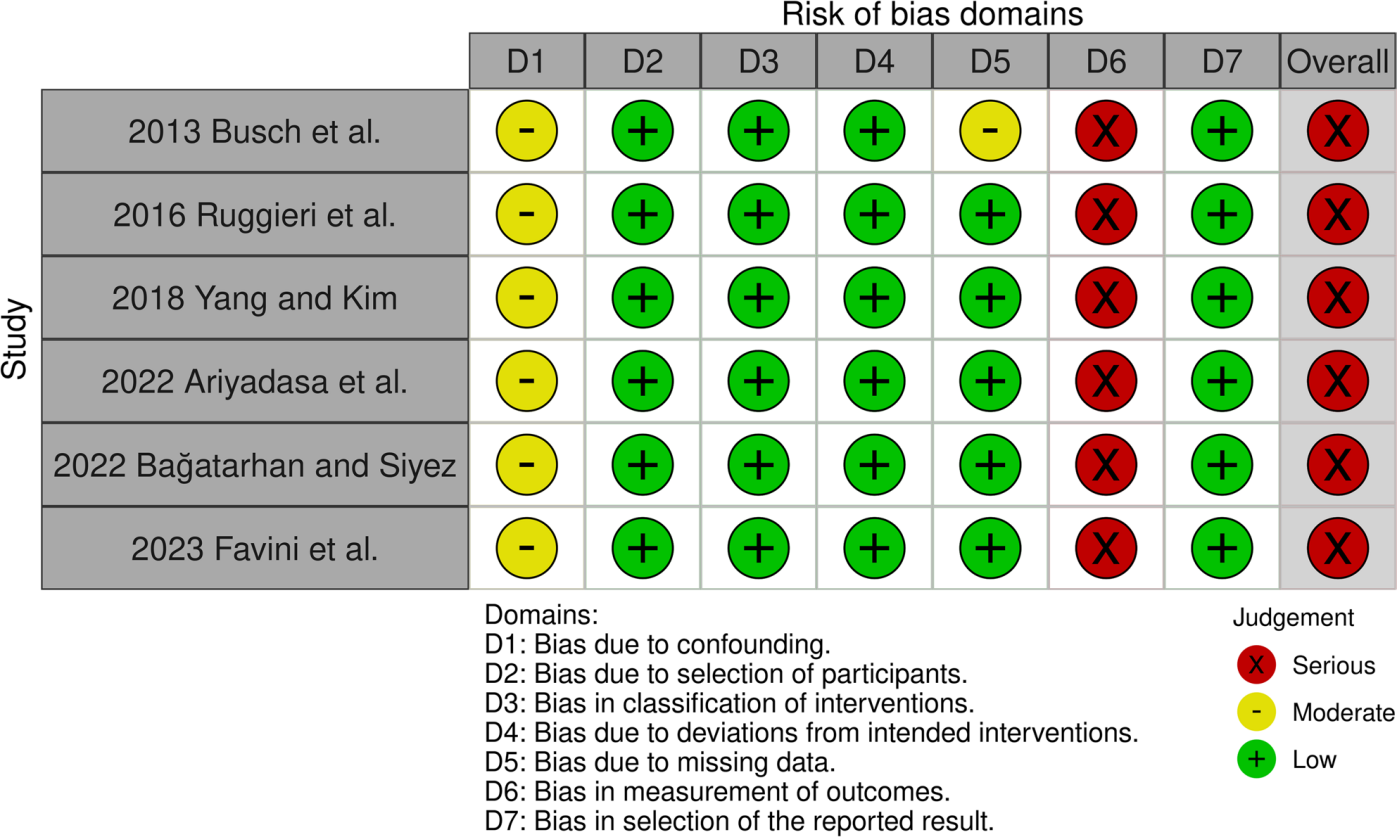
Risk of bias assessment of the non-randomised studies using the ROBINS-I tool. Judgements are shown for each domain (D1–D7) and overall. Green = low risk; yellow = moderate risk; red = serious risk

When assessing the risk of bias, for randomised studies, the items ‘Blinding of participants and personnel’ and ‘Blinding of outcome assessment’ obtained a ‘high’ risk of bias in all studies, while ‘allocation concealment’ was predominantly classified as ‘unclear’. For the ‘Incomplete outcome data,’ ‘Selective reporting,’ and ‘other bias’ categories, the majority obtained a ‘low’ risk of bias (Fig. 2). As one of the domains considered relevant was classified as ‘high’ in each randomised study, the overall risk of bias for each trial was therefore considered ‘high’. For non-randomised studies, the overall risk of bias was assessed as ‘serious’, with the category ‘Bias in measurement of outcomes’ showing the highest risk of bias (Fig. 3).

### Meta-analysis results

A meta-analysis was performed using a random-effects model, since the indicators of homogeneity (I² < 50% and Chi² test *p* > 0.05) were not met, to assess the ‘Internet addiction’ variable by entering the relevant arithmetic data at the first assessment point following the end of the intervention. Subgroups were established based on whether parents were included in the intervention (‘Interventions for adolescents and their parents’ and ‘Interventions only for adolescents’, respectively). Thus, the forest plot shows an overall effect for the ‘Internet addiction’ variable and two independent results for each of the subgroups analysed.

Two studies with an experimental design were included [46, 49] as well as four studies with a quasi-experimental one [53, 55, 57, 58]. A total of 859 participants were evaluated, with the smallest sample size being 30 individuals [53] and 570 the largest [57].

**Fig. 4:**
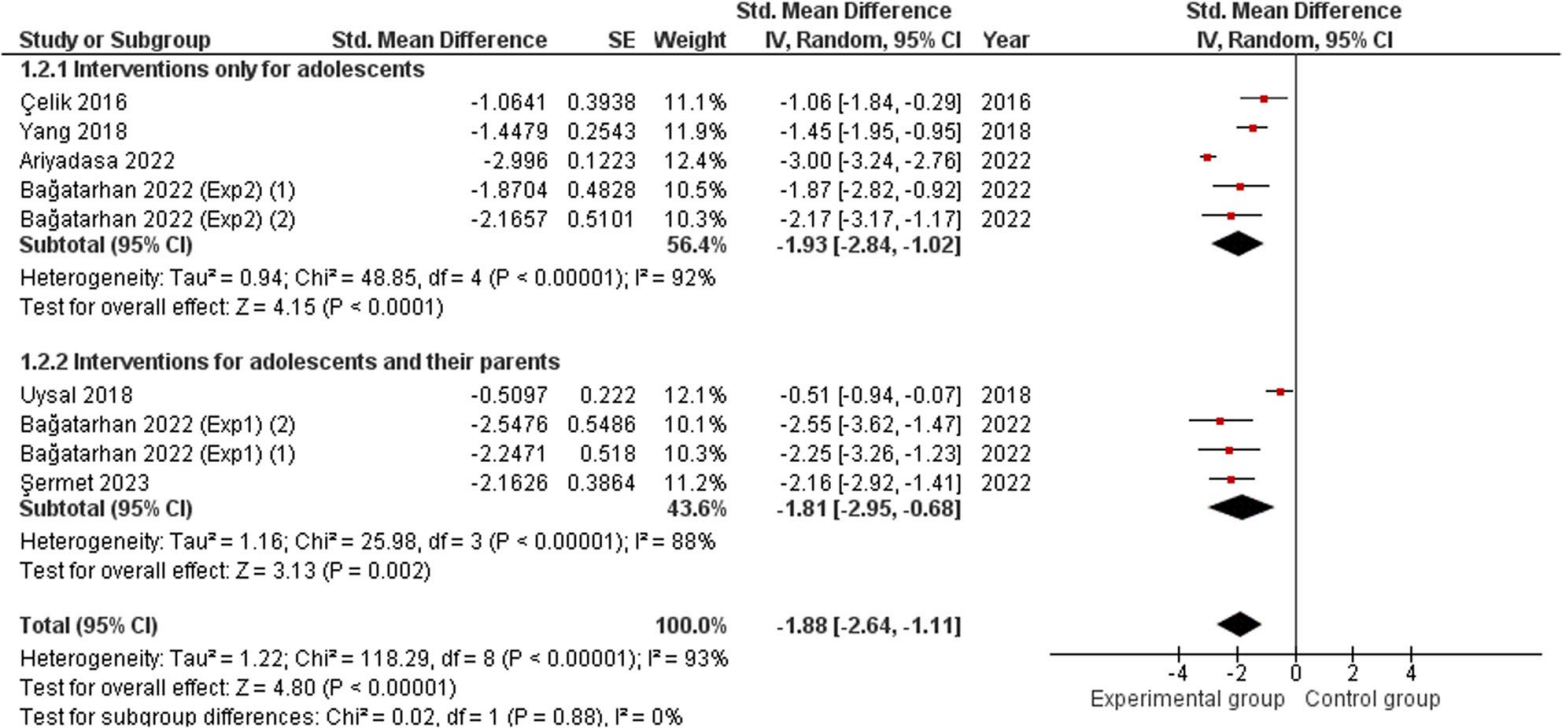
Forest plot of the meta-analysis conducted to assess the ‘Internet addiction’ variable

Statistically significant differences were noted in favour of the experimental group compared to the control group in terms of improvement in the ‘Internet addiction’ variable, both in the overall analysis (SMD = − 1.88; 95% CI [− 2.64, − 1.11], *p* < 0.00001) and in the subgroup analysis: in favour of the ‘Interventions only for adolescents’ subgroup (SMD = − 1.93; 95% CI [− 2.84, − 1.02], *p* < 0.0001), and in favour of the subgroup ‘Interventions for adolescents and their parents’ (SMD = − 1.81; 95% CI [− 2.95, − 0.68], p = 0.002). In all cases, the SMD was greater than 0.8, so the magnitude of the effect was classified as a ‘large effect’. The forest plot for the meta-analysis is shown in Fig. 4.

### Risk of publication bias and sensitivity analysis

Testing for risk of publication bias using the funnel plot asymmetry test depends on the number of studies, where less than 10 studies result in low power [60]. For this reason, this test was not included in this review as the minimum threshold of 10 studies was not met. The sensitivity analysis showed no significant changes in the global effect when each of the comparisons included in the meta-analysis was individually eliminated (Table 2).

**Table 2:** Sensitivity analyses based on various exclusion criteria for the ‘Internet addiction’ variable

Excluded comparison	No. of Comparisons	SMD (95% CI)	*P* for SMD	I^2^	*P* for heterogeneity
Çelik 2016 [53]	8	−1.98 (−2.80, −1.16)	< 0.00001	94%	< 0.00001
Yang 2018 [55]	8	−1.94 (−2.81, −1.06)	< 0.0001	94%	< 0.00001
Ariyadasa 2022 [57]	8	−167 (−2.23, −1.12)	< 0.00001	77%	< 0.00001
Bağatarhan 2022 [58] (Exp2) (1)	8	−1.88 (−2.71, −1.04)	< 0.00001	94%	< 0.00001
Bağatarhan 2022 [58] (Exp2) (2)	8	−1.84 (−2.68, −1.01)	< 0.0001	94%	< 0.00001
Uysal 2018 [46]	8	−2.07 (−2.69, −1.44)	< 0.00001	86%	< 0.00001
Bağatarhan 2022 [58] (Exp1) (2)	8	−1.80 (−2.63, −0.97)	< 0.0001	94%	< 0.00001
Bağatarhan 2022 [58] (Exp1) (1)	8	−1.83 (−2.67, −1.00)	< 0.0001	94%	< 0.00001
Şermet Kaya 2023 [49]	8	−2.20 (−2.37, −2.02)	< 0.00001	94%	< 0.00001

*SMD* standardised mean difference, *CI* Confidence Interval

### Certainty of the evidence

The certainty of the evidence was rated as ‘low’ due to the risk of bias in the studies, as they were both experimental and quasi-experimental. Yet, as the results obtained showed a large effect size, the results were rated as ‘important’ (Table 3).

**Table 3:** Certainty of evidence according to the GRADE tool for the ‘Internet addiction’ variable

Certainly assessment							No. of patients		Effect		Certainty	Importance
No. of studies	Study design	Risk of bias	Inconsistency	Indirect evidence	Imprecision	Others	Interventi-on	Compa-rison	Relative (95% CI)	Absolute(95% CI)		
Internet addiction												
6	CTs	Very serious^a^	Not serious	Not serious	Not serious	Strong association	422	437	-	SMD **−1.88 **(−2.64 to −1.11)	⨁⨁◯◯LOW	IMPORTANT

*Abbreviations*: *CTs* Clinical Trials, *CI* confidence interval, *SMD* standardised mean difference

^a^ The included studies show high risk of bias

## Discussion

The distinctive contribution of this review is its focus on health education interventions in adolescents. Earlier reviews have made valuable contributions by examining a wide range of strategies or by emphasising prevention in school settings. Our synthesis builds on this body of work by concentrating on health education at this stage of development, providing a clearer understanding of how these interventions can influence Internet addiction, time spent online, and, with more limited impact, social media addiction.

The included studies ranged from experimental to quasi-experimental designs, and the interventions were heterogeneous. In our meta-analysis, we also examined subgroups with and without parental involvement.

Among the interventions that showed positive outcomes, effective approaches encouraged healthier patterns of Internet use and emphasised participatory and skills-building activities with adolescents, including role-play, group discussions and brainstorming. These interventions were often delivered in educational settings or addressed broader health promotion topics, reflecting the WHO definition of health education, which goes beyond the transfer of information to build motivation, skills and confidence to support healthier choices. It also connects with the WHO concept of life skills, described as abilities to cope with everyday challenges. In this context, these include decision-making, problem-solving and self-awareness [28]. Developing these abilities may help adolescents to reflect on their Internet use and move towards healthier patterns of behaviour. In line with our findings, a school-based prevention review on Internet addiction in adolescents indicated that prevention interventions should address related health problems and develop common goals such as refusal skills and increasing awareness. It also noted that risk behaviours appear to be interrelated, which has led to calls for more holistic approaches in prevention. This perspective is consistent with the participatory and skills-building strategies identified in the health education interventions that showed significant outcomes in the present review [30]. However, because that review focused on school-based prevention approaches, our work adds by examining health education interventions that also extend beyond prevention.

In our review, most studies showed improvements in Internet addiction, suggesting that health education interventions can make a positive difference. A previous systematic review of school-based interventions for prevention also found reductions in Internet addiction scores, particularly when interventions promoted healthy habits and behaviours in Internet use. Our findings complement this evidence, as the interventions included in this review tended to promote more balanced Internet use, with benefits extending beyond prevention and supporting adolescents in developing skills such as self-awareness [61].

Evidence on social media addiction was scarce, with only two studies addressing this outcome. One reported improvements [59], while the other showed less time spent on social media but no change in addiction scores, which may still point to a preventive effect even if addiction itself did not improve [45]. A recent narrative review that described interventions for problematic social media use and its impact on adolescent mental health also noted that school-based strategies can help by reaching adolescents at a key stage and giving them tools for healthier social media habits [31]. The long-term and wider effectiveness of these interventions remains uncertain, and our review identified the same gap.

In this review, the majority of studies that analysed Internet usage time employed different types of questionnaires and focused on various aspects, such as general Internet usage time during the week or at weekends, or time spent online playing video games or on social media. For this variable, most of the studies analysed found significant improvements in at least one of the assessments carried out. However, the variability in both the questionnaires used and the results obtained suggests that the effects of health education interventions may differ depending on the specific context.

As for those studies that included parents in the intervention, the results showed overall significant improvements in at least one of the variables. This is consistent with the conclusions of another systematic review with a similar approach, which recommends involving parents and other relevant agents in the prevention of Internet addiction [21].

Although the content of the intervention aimed at parents varied, evidence from some interventions suggests that strengthening parent–child relationships or offering support through home visits also yielded positive effects [49, 58]. These findings are consistent with the results of a meta-analysis that examined parenting styles and their correlation with Internet addiction, showing a positive correlation between negative parenting styles and adolescents with problematic Internet use and a negative correlation with positive parenting styles [62]. Thus, involving parents with the aim of improving their relationship with their children or conducting home visits could have indirectly influenced parenting styles with regard to Internet use patterns.

The findings of the systematic review are consistent with the results of the meta-analysis conducted for the ‘Internet addiction’ variable. For the overall meta-analysis, a statistically significant difference in favour of the intervention was found, with a large effect size. Looking at the subgroups, interventions aimed only at adolescents as well as those that also included parents showed statistically significant improvements with a large effect size. These results are in line with another similar meta-analysis that assessed the effects of non-pharmacological interventions on Internet addiction in young people with Internet addiction. In the subgroup meta-analysis, they found that educational interventions were effective in reducing levels of Internet addiction, with an effect size considered large according to conventional thresholds. However, despite the numerous studies that examined different intervention strategies, only two were included in the educational intervention category of the subgroups [32], which could be influenced by the paucity of studies in the literature with an experimental design. Our review refines this evidence by focusing on educational interventions, expanding the number of studies analysed, and providing further evidence that their effects are favourable across different contexts.

In the assessment of the strength of the evidence in the overall meta-analysis using the GRADE tool, the importance was classified as ‘important’ and the certainty as ‘low.’ Because both experimental and quasi-experimental studies were included, the certainty of the evidence was downgraded to avoid overestimating the findings. This indicates that, while the direction of the results appears favourable, the certainty remains low and more high-quality experimental studies are needed to confirm these benefits.

In terms of risk of bias, the most common type was related to the blinding of participants and evaluators, which was associated with the assessment of the results. However, this is an inherent risk in educational interventions, as participants cannot be blinded to whether they are receiving education. Furthermore, blinding was not possible during evaluation because the participants completed the questionnaires themselves.

A limitation of this review is that it analysed studies with experimental and quasi-experimental designs. Although this provides a broader view of the types of interventions carried out and their results, it may also entail a higher risk of bias during the analysis. Therefore, being aware of this limitation, the certainty of evidence obtained in the GRADE tool was downgraded. Another limitation was the variability among the interventions analysed in terms of their content, frequency, and duration, as well as in relation to the variety of instruments used to measure the variables. Similarly, heterogeneity has been reported in a school-based prevention review, which highlighted variability in scope and assessment tools, along with the absence of standardised diagnostic criteria and the use of self-reported data. This suggests that such heterogeneity is a persistent challenge in the field [30]. Finally, relatively few studies were included that examined the effects on social media addiction.

Overall, the findings of this review suggest that health education interventions can play a valuable role in helping adolescents and their families both address problematic online behaviours and encourage healthier patterns of Internet use. The evidence is still developing, but the general direction of results is encouraging.

## Conclusions

This study suggests that health education for adolescents can be effective in reducing Internet addiction, Internet use time, and, to a more limited extent, social media addiction. Although the interventions analysed were heterogeneous, several recurring features could be identified, including participatory and skills-building strategies such as role-play, group discussions and brainstorming, specific content on Internet use and addiction, delivery in educational settings, and the inclusion of broader health promotion topics. These findings support the potential applicability of health education in diverse public health and educational contexts. Nevertheless, the certainty of the evidence is low, and further experimental studies with greater standardisation are needed to consolidate this evidence.

## Data Availability

The datasets used and/or analysed during the current study are available from the corresponding author on reasonable request.

## Abbreviations

BSMAS: Bergen Social Media Addiction Scale
CG: Control Group
CI: Confidence Interval
CINAHL: Cumulative Index to Nursing and Allied Health Literature
CTs: Clinical Trials
CIUS: Compulsive Internet Use Scale
IA: Internet Addiction
IAS: Internet Addiction Scale
IAT: Internet Addiction Test
ISS: German Internet Addiction Scale
EG: Experimental Group
GRADE: Grading of Recommendations Assessment, Development and Evaluation
K-scale: Internet Addiction Proneness Scale
PIUS: Problematic Internet Use Scale
PMC: PubMed Central
PCIAS: Parent–Child Internet Addiction Scale
PRISMA: Preferred Reporting Items for Systematic reviews and Meta-Analyses
OCS: Online Cognition Scale
ROBINS-I: Risk Of Bias In Non-randomised Studies - of Interventions
SMA: Social Media Addiction
S-MAT: Social Media Addiction Test
SMD: Standardised mean difference
WoS: Web of Science
